# Supercritical Impregnation of Mesoglycan and Lactoferrin on Polyurethane Electrospun Fibers for Wound Healing Applications

**DOI:** 10.3390/ijms24119269

**Published:** 2023-05-25

**Authors:** Stefania Mottola, Gianluca Viscusi, Giovanna Iannone, Raffaella Belvedere, Antonello Petrella, Iolanda De Marco, Giuliana Gorrasi

**Affiliations:** 1 Department of Industrial Engineering, University of Salerno, Via Giovanni Paolo II, 132, 84084 Fisciano, Italy; smottola@unisa.it (S.M.); gviscusi@unisa.it (G.V.); g.iannone55@studenti.unisa.it (G.I.); ggorrasi@unisa.it (G.G.); 2 Department of Pharmacy, University of Salerno, Via Giovanni Paolo II, 132, 84084 Fisciano, Italy; rbelvedere@unisa.it; 3 Research Centre for Biomaterials BIONAM, University of Salerno, Via Giovanni Paolo II, 132, 84084 Fisciano, Italy

**Keywords:** supercritical carbon dioxide, electrospinning, transdermal drug delivery, release tests modeling, cytotoxicity tests

## Abstract

Fibrous membranes of thermoplastic polyurethane (TPU) were fabricated through a uni-axial electrospinning process. Fibers were then separately charged with two pharmacological agents, mesoglycan (MSG) and lactoferrin (LF), by supercritical CO_2_ impregnation. Scanning Electron Microscopy (SEM) and Energy Dispersive X-ray Spectroscopy (EDS) analysis proved the formation of a micrometric structure with a homogeneous distribution of mesoglycan and lactoferrin. Besides, the degree of retention is calculated in four liquid media with different pHs. At the same time, angle contact analysis proved the formation of a hydrophobic membrane loaded with MSG and a hydrophilic LF-loaded one. The impregnation kinetics demonstrated a maximum loaded amount equal to 0.18 ± 0.20% and 0.07 ± 0.05% for MSG and LT, respectively. In vitro tests were performed using a Franz diffusion cell to simulate the contact with the human skin. The release of MSG reaches a plateau after about 28 h while LF release leveled off after 15 h. The in vitro compatibility of electrospun membranes has been evaluated on HaCaT and BJ cell lines, as human keratinocytes and fibroblasts, respectively. The reported data proved the potential application of fabricated membranes for wound healing.

## 1. Introduction

Skin is a natural barrier that protects humans from the external environment, so it is important to take care of it and know how to manage wounds that may occur [1,2]. The skin itself has good regenerative properties by which wound healing happens. This is often not enough due to the spread of the infection to healthy tissue, which lengthens the healing time. In such cases, wound dressings have to be used; particularly, they must protect the wound from further trauma, ensure a moist environment, absorb or remove excess exudate, and prevent infections by promoting healing [3,4]. The research for new pharmaceutical pathways that promote the repair of the epithelium is very active. Some studies have focused attention on the effective re-epithelializing action of sodium mesoglycan (MSG) and on the unexpected properties of lactoferrin (LF) that contribute to wound repair [5,6]. MSG is a biomaterial containing natural glycosaminoglycans (GAG) like heparan-, dermatan-, chondroitin-sulfate, and low molecular weight heparin. MSG is an attractive drug because it affects the antithrombotic pathways, but it is also useful in cases of cerebral vascular disease and chronic venous ulcers. MSG is generally taken through the oral or the parenteral route but provides long therapy times with high and repeated doses that cause side effects; thus, developing topical devices is presented as a solution [7,8]. LF is an iron-binding glycoprotein present in body fluids (saliva, tears, and mucous secretions) synthesized by glandular epithelial cells [9]. Thanks to its biological activities, such as elevation of basal metabolic rate and anti-nociceptive and anti-oxidative stress activities, LF is used in therapy against obesity and anti-aging but also has a promising role in treating terminal cancer. It is susceptible to proteolytic enzymes, so it is quickly eliminated from the blood that involves numerous administrations when taken by the parenteral pathway. The use of delivery systems is one of the approaches to improve its utility [10]. Both sodium mesoglycan and lactoferrin enhance the re-epithelialization process by encouraging the proliferation and migration of dermal fibroblasts and human epidermal keratinocytes [11]. Moreover, lactoferrin supports the host’s defenses thanks to its antibacterial, bacteriostatic, antiviral, and antifungal activities [8,10,12]. Appropriate polymeric support must be designed to deliver these active pharmaceutical compounds. Among all, polyurethane is one of the most used polymers to produce dressing such as patches thanks to its properties suitable for the purpose: high biocompatibility, durability, tear or wear resistance, elasticity, sterility, and propensity to healing [13]. The drug delivery method is one of the main challenges linked to the development of new pharmaceutical systems because it affects the occurrence of side effects or the effectiveness of treatment. Drug delivery impacts various factors, such as pharmacokinetics, distribution, pharmacodynamics, metabolism, and toxicity [14,15,16]. The use of polymeric nanofibers, based on biocompatible and biodegradable polymers, is an attractive option to overcome these limits thanks to the controlled and uniform delivery given by the high specific surface area. Moreover, these systems meet the requirements of flexibility, high porosity, and good protection from the external environment necessary for a suitable dressing [17,18]. Among the different techniques able to obtain nanofibers, electrospinning has been a widely attractive and used process for nanofibers production in the last 20 years because it is simple, continuous, reproducible, and scalable [19]. Moreover, it is the only method that provides an optimal morphology for the application of interest [20,21,22]. The topical devices currently on the market have some drawbacks linked to the rapid release and the low loading of the active principle. For example, Franco et al. succeeded in impregnating mesoglycan on calcium alginate. The produced system allows for a fast and complete drug release, which is suitable for efficient and prompt action on serious injury [12]. Kataria et al. used polyvinyl alcohol loaded with sodium alginate nanofibers and ciprofloxacin as a transdermal patch for the release of an antibiotic drug. The designed system ensured a tuned and continuous release in about 7 h, while the in vitro activity ensured a controlled release from the transdermal patch [1]. Akduman et al. produced ultra-fine fiber mats of thermoplastic polyurethane containing naproxen by electrospinning. Their goal is to evaluate the effect of naproxen loading as well as the thicknesses of the membrane on in vitro release, demonstrating that as the thickness increases, the total released drug decreases, allowing for extending the release time up to 120 h [23]. In some other previous works, other kinds of thermoplastic polyurethane (TPU)-based medical devices were produced [24,25,26,27]. The impregnation process assisted by supercritical carbon dioxide (scCO_2_) allows overcoming these obstacles thanks to the possibility of having, at the same time, high diffusivity, near-zero surface tension, high density, and high solvent power. For these reasons, the solvent residues eventually present in the patches are removed as the affinity of the used organic solvents with CO_2_ is very high; therefore, the supercritical process allows to have in a one-step process the impregnation of active principles at mild process conditions without requiring the use of organic solvents. Then again, the promotion of the recovery of the solvent used in the production of the polymeric material, the non-use of organic solvents, and the employment of lower process times with respect to traditional techniques make it a process of great interest to produce pharmaceutical systems [28,29,30]. Supercritical fluid impregnation (SFI), a process based on the use of scCO_2_, has been successfully used to obtain medical devices that can be used for wound healing [12,31,32]. In this work, SFI has been used to impregnate sodium mesoglycan and lactoferrin on the produced TPU electrospun matrices to develop two types of composite systems (TPU/MSG and TPU/LF) as topical devices to be used for the wound healing. The optimum operating conditions were selected in order not to compromise the properties of the patch; then, the chosen conditions were used for the supercritical impregnation of the active principles into the polymeric matrices. The produced patches have been characterized to test the mechanical and liquid retention properties; also, the release of the drug impregnated on the patches was performed, and the mathematical model of the release curves was studied. Finally, the impregnated patches have been tested on cell cultures to define their effect. Human keratinocytes and fibroblasts were used, and it has been evaluated for their activity in the main processes involved in correct tissue regeneration.

## 2. Results

### 2.1. Production of Electrospun Fibers

**Figure 1:**
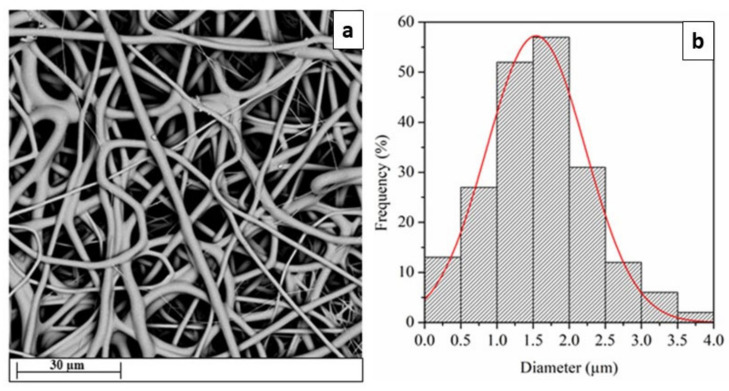
SEM image (**a**) and diameter distribution (**b**) of neat TPU electrospun fibers.

After optimizing the electrospinning conditions to obtain beads-free fibrous membranes, neat TPU fibers were successfully obtained (Figure 1).

The neat TPU fibers appeared to possess a defect-free random oriented fibrous structure with a mean diameter of 1.58 ± 0.20 µm. The fibrous mats did not show a nanometric morphology, but since the targeted application, smooth, homogeneous, and bead-less nanofibers structure has a more significant effect than the fiber diameter [33].

### 2.2. Supercritical CO_2_ Contact Tests

Before the active ingredient’s impregnation, it was necessary to carry out tests to evaluate the behavior of the polyurethane fibers when in contact with CO_2_ in supercritical conditions at different values of temperature and pressure. Films of 1 cm × 1 cm were cut and placed inside a steel sample holder. The T-P pairs investigated were the following: T = 40 °C, P = 15 MPa; T = 40 °C, P = 17 MPa; T = 50 °C, P = 15 MPa; T = 50 °C, P = 17 MPa. The samples obtained by operating at pressures of 17 MPa, independently of the chosen temperature, proved to be considerably stiffer at the end of the process. Given the potential industrial application of the plasters produced, it is necessary to maintain the initial flexibility. Based on a macroscopic evaluation of the different samples, after 24 h of contact, it was decided to work in conditions of T = 50 °C and P = 15 MPa to guarantee optimal solubilization of the active principle. Even the depressurization speed has shown a significant influence on the flexibility of the structure: it is advisable to decrease the pressure inside the system in a slow and controlled way to limit the stiffening of the polymer chains with consequent loss of the characteristic flexibility of the plasters. For this reason, the depressurization rate used is 5 bar/min.

### 2.3. Supercritical Impregnation of Active Compounds into the Electrospun Fibers

**Figure 2:**
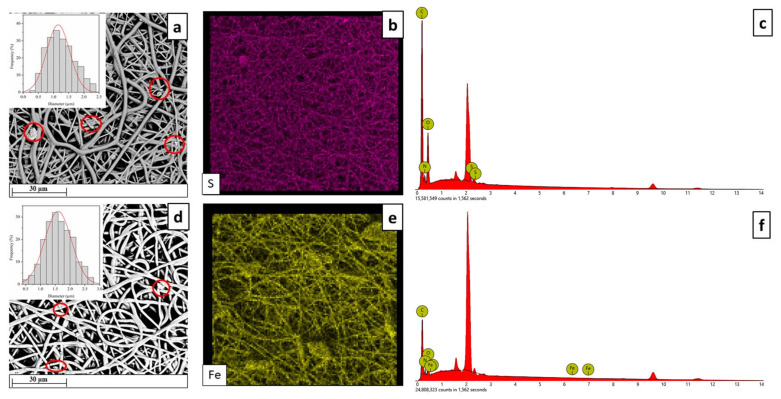
SEM micrographs, diameter distribution, and EDS analysis of MSG-TPU (**a**–**c**) and LF-TPU (**d**–**f**) electrospun fibers.

The impregnation kinetics were determined by varying the contact time (from 2 to 48 h) at a fixed pressure of 15 MPa, and temperature of 35 °C. After the impregnation, the morphology of the TPU electrospun membranes loaded with the active compounds was analyzed. Figure 2 reports the SEM micrographs, the diameter distribution, and the elemental mapping on electrospun fibers.

**Figure 3:**
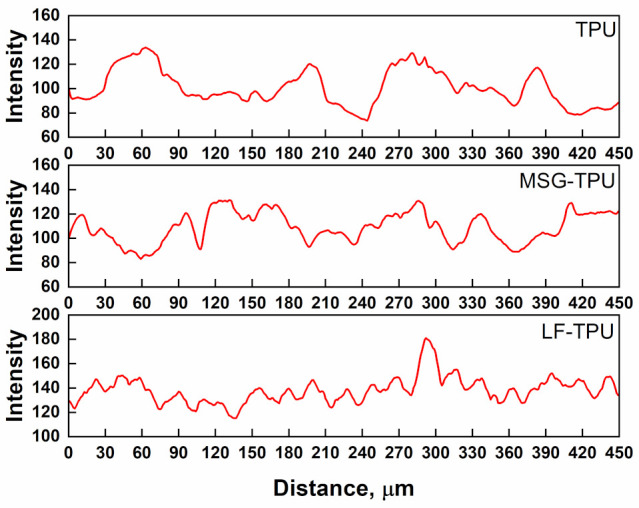
Profile plots of electrospun systems.

The distributions reported in Figure 2 confirm that the presence of the drugs did not compromise the morphology of TPU fibers. No noticeable differences could be observed compared to the pristine TPU fibers, nor the presence of defects, beads, or inhomogeneous structures. The mean diameter changes from 1.58 µm for neat TPU to roughly 1.60 µm for MSG-TPU and LF-TPU electrospun fibers, respectively. Moreover, few agglomerates are visible, which allows for proving the presence of active drugs on the TPU fibers. To confirm the distribution of drugs on the polymeric structure, EDS analysis was carried out, and the elemental maps are reported in Figure 2c (sulfur) and 2f (iron) belonging to MSG and LF, respectively. The analysis of SEM micrographs allowed for studying the profile plots (intensity vs. distance) (Figure 3). 

**Figure 4:**
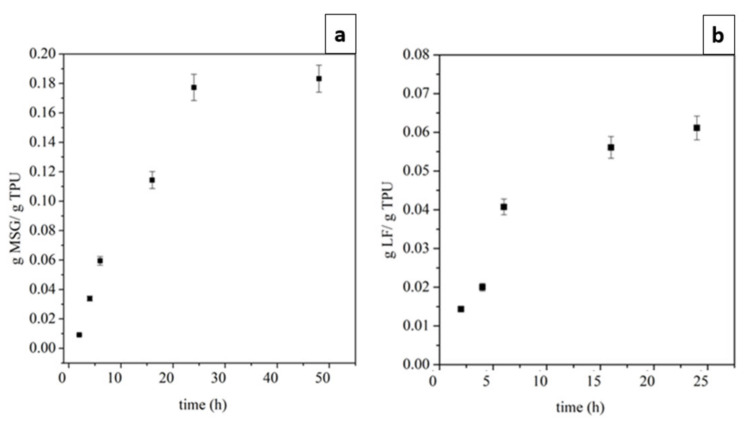
Impregnation kinetics of MSG (**a**) and LT (**b**) on TPU.

As evidenced by Figure 3, the surface topography clearly differs in height. Indeed, in addition to incorporating the two drugs, the fibers’ arrangements and surface properties can undergo substantial modifications and affect the nanofiber surface roughness. A change in profile height after loading MSG and LF was observed. The R_a_ values are 97, 107, and 141, while RMS values are 98, 107, and 141 for TPU, MSG-TPU, and LF-TPU, respectively. So, the impregnation of the two drugs led to an increase in surface roughness which could be considered a crucial value for cell differential and proliferation [34]. The effective quantity of impregnated MSG and LT in the polymeric matrices was evaluated by UV/vis spectrophotometry. The loadings are reported in Figure 4a and b for MSG and LT, respectively. It is shown how the quantity of impregnated active principles increases with the time of contact up to reach a maximum value equal to 0.18 ± 0.20% and 0.07 ± 0.05% for MSG and LT, respectively.

### 2.4. Contact Angle

**Figure 5:**
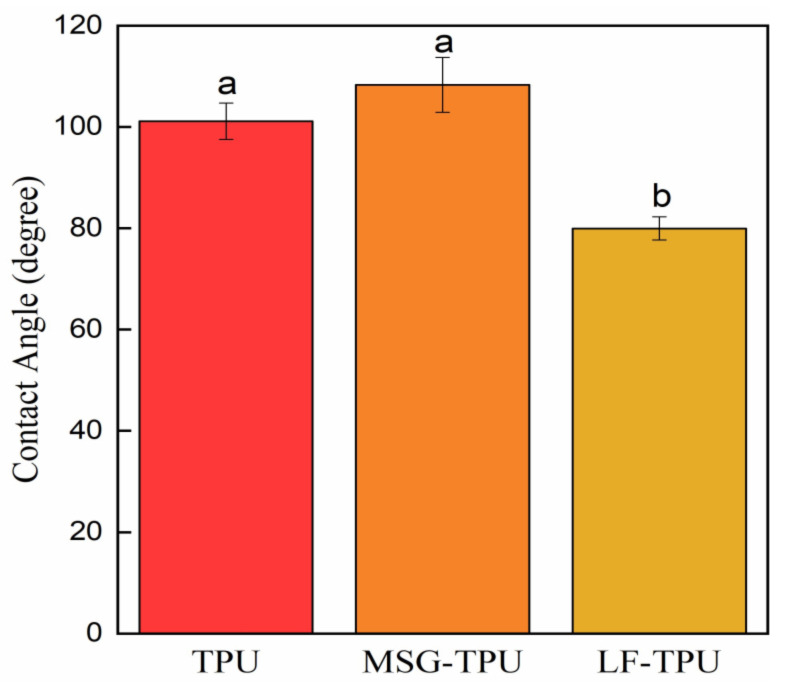
Water contact angle of supercritical impregnated TPU electrospun fibers. For each fiber, different superscript letters in the same bar indicate that the mean values are significantly different (*p* ≤ 0.05).

The hydrophobicity analysis was carried out on the electrospun membranes since wettability is a crucial factor in biocompatibility [35]. Values of contact angles are reported in Figure 5.

Pristine TPU electrospun membrane showed high CA values (CA ≥ 100°). It indicates that the water droplet does not easily spread on the surface since the intrinsic hydrophobicity of the nonwoven organization of the membrane, attributable to the micro/nanofiber morphology, is responsible for the substantial decrease in the wettability of these materials. High hydrophobicity is desirable for wound healing applications since it is one of the factors affecting cell proliferation. As can be seen from Figure 5, the contact angles change from 100° to 110° and 80° for TPU, MSG-TPU, and LF-TPU, respectively. The impregnation of MSG could contribute to reducing the free volume and the pore size. So, the spreading of water drops is limited. The lower CA value for the LF-TPU sample might be attributed to the steric volume of LF, which might create spaces among the randomly distributed fibers, keeping the fibers apart and resulting in better water droplet infiltration. The increase in surface roughness for MSG-TPU might justify the increase in CA while, despite the increase in surface roughness after the loading of LF, the lower CA of LF-TPU might be due to the steric hindrance of the globular protein and the consequent increase in free volume. 

### 2.5. Liquid Retention Tests

**Figure 6:**
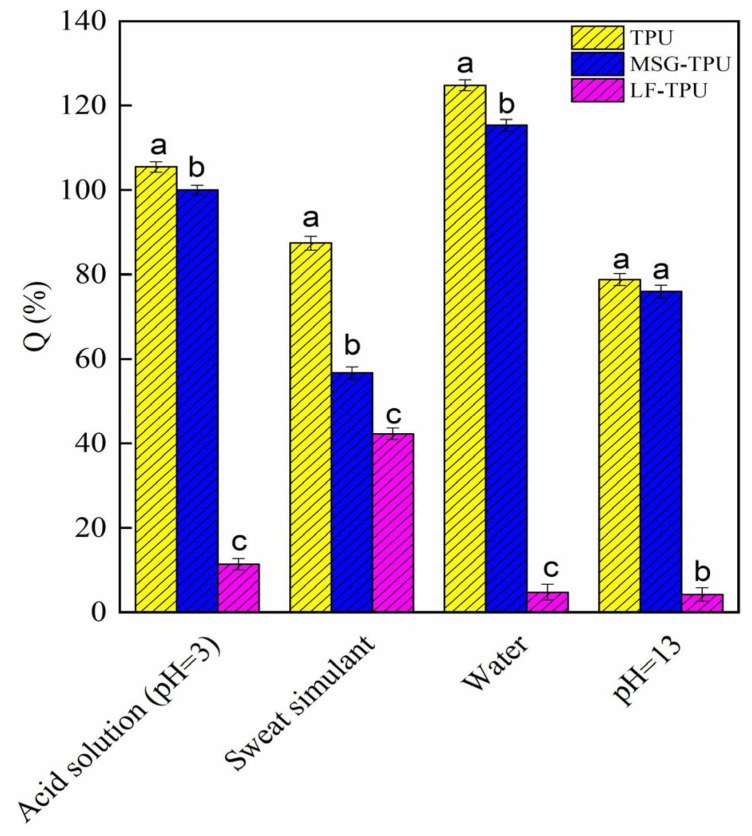
Liquid retention tests of the electrospun membranes. For each fiber, different superscript letters in the same bar indicate that the mean values are significantly different (*p* ≤ 0.05).

Figure 6 reports the retention degree (Q%) of electrospun mats.

The data cannot be easily described since many parameters, such as roughness, porosity, polar surface sites, and point of zero charges, should be considered. Concerning the water retention test, the pristine TPU shows a retention degree of about 125%, which decreases to 115% for MSG-TPU and 5% for LF-TPU. For water absorption (pH = 7), it is possible to observe that the TPU sample shows a greater absorption capacity due to the high surface roughness and the high degree of porosity. In a neutral environment, the high degree of retention of the electrospun TPU sample can be ascribed to the characteristic porosity of the fibrous system. The presence of mesoglycan causes a slight variation in retention degree; however, the LF-TPU system showed a Q (%) of approximately 5%. This value can undoubtedly be linked to the high steric hindrance of the LF (globular protein), which could reduce the free volume between the fibers and the increased surface roughness, resulting in a reduction in the amount of water absorbed. At higher pH, weak interactions are likely to occur between the amino groups of adjacent urethane chains, thus contributing to forming of an interconnected network [36]. This phenomenon could therefore justify the reduction of the absorption capacities of the samples subjected to tests. This trend is also reflected in acidic conditions (pH = 3). However, the greater absorption capacity in acidic conditions can be correlated with the presence of H^+^ ions in the solution. In fact, for pH values lower than the isoelectric point of the absorbent system, the presence of H^+^ ions induces a protonation of the surface groups of the system, which expose a high number of positive surface charges. The presence of these charges causes an electrostatic repulsion between the polymeric chains of the network, thus determining an increase in the free volume. Finally, the same trend is again highlighted among the samples in an environment simulating sweat. In this case, however, it is necessary to consider other phenomena, such as the ionic strength due to the presence of salts and the point of zero charge of the system. The sweat-simulating solution has a pH of approximately 5.8, close to the zero charge point of the electrospun polyurethane membrane; this implies that in this pH range, the sum of the positive charges on the surface is equal to the sum of the negative charges. The membrane, therefore, has a net charge equal to zero, and water absorption is due overall to the diffusion of water molecules in the polymeric network. However, the high absorption capacity of the LF-TPU system (about 43%) differs considerably from the values obtained from the other conditions; this could be due to the chemical nature of lactoferrin, a quaternary protein naturally present in biological liquids such as tears and sweat, which in the presence of saline solutions rich in salts could be quickly released from the polymeric fiber. Therefore, the diffusion of lactoferrin towards the aqueous medium could favor the formation of new pores and active sites for water absorption, justifying the high value detected.

### 2.6. Release Tests and Mathematical Modeling

**Figure 7:**
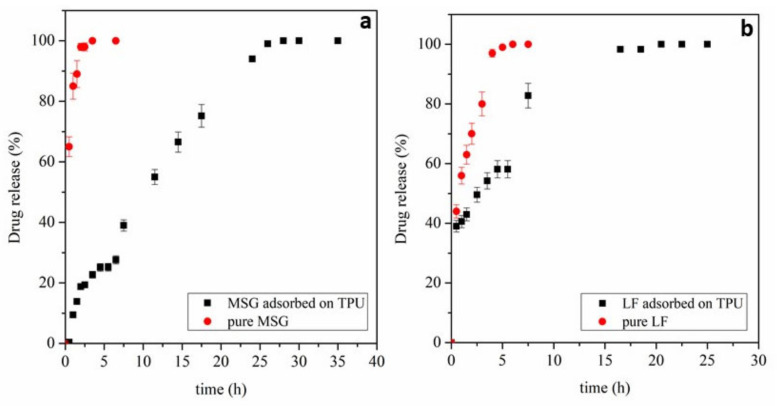
Release profiles of (**a**) MSG-TPU and (**b**) LF-TPU in PBS medium.

After the impregnation of drugs, in vitro release tests were also carried out to compare the release times of the drugs in a PBS solution at pH = 7.4 using a Franz diffusion cell to simulate the contact with the human skin. The samples were analyzed through a UV/vis spectrophotometer using the previously constructed calibration lines. Release profiles of MSG and LF are reported in Figure 7.

As it is possible to observe in Figure 7a, the release of unprocessed mesoglycan in the liquid medium is speedy. It is completed in about 2.5 h, while it is delayed using TPU electrospun on which the active principle has been adsorbed using supercritical CO_2_. Specifically, the release of MSG reaches a plateau after about 28 h. A burst effect of around 25% can be observed after 5.5 h. In the first phase, therefore, there is probably the release of the molecules present on the surface; instead, in the second phase, the mesoglycan incorporated in the polymeric matrix can diffuse toward the surface. The liquid medium must diffuse into the film, solubilizing the mesoglycan contained therein and letting MSG diffuse toward the external medium. This phenomenon explains why the release can be considerably prolonged. In Figure 7b, it is possible to observe what instead happens when the active principle used is LF. After about 6 h, the unprocessed lactoferrin is wholly dissolved in the liquid medium; when it is absorbed on the surface of the polyurethane, the release is prolonged up to about 20 h. Although after one hour, 40% of the drug is released while the remaining 60% is reached after 5 h, which is a symptom of the fact that a high amount of the molecules is present on the surface, the presence of the remaining part inside the polymeric matrix favors a slower release, allowing the fibrous system to be functional for longer times. The mathematical modeling of the release data was carried out to know the mechanisms that influence the releases of the two drugs under examination. It has been observed that for the tested different systems, it is possible to describe the release data through a second-order model known as Ho’s equation (Equation (1)):

(1) $$\frac{t}{q_{t}}=\frac{1}{{k*q}_{e}^{2}}+\frac{t}{q_{e}}$$

where K is the release kinetic constant, q_e_ is the equilibrium released drug amount, and q_t_ is the release drug amount at a specific time t. Regarding the impregnated TPU membranes, two phenomena are considered to explain the presence of the double pass. The first step, represented by the K_1_ constant, is attributable to the drug molecule’s diffusion from the polymer’s surface. In contrast, the second step, represented by K_2_, is linked to the molecular relaxation of the polymer, which allows the drug trapped between the fibers to be released. The mathematical modeling provides for obtaining the kinetic constants for each studied system which have been reported in Table 1.

Indeed, observing the values of the kinetic constants in the case of pure drugs, it is expected that the diffusion mechanism of lactoferrin is slower than that of mesoglycan, due probably to the thicker fibers as well as the steric hindrance of the globular protein, which could contribute to slow down the diffusion along the fibrous system, confirming the trend obtained. Since the necessity of considering the double-step model, the release profiles of MSG-TPU and LF-TPU have been divided into two parts, linked respectively to the two steps. Moreover, in the case of the TPU+MSG system, it is possible to observe that an initial induction time is present (time necessary to overcome the resistance to transport so that the mesoglycan begins to diffuse). Apart from that, in both cases, the step linked to the diffusion of the drug from the surface has higher kinetic constants than the second step: this explains the presence of the initial burst, which is higher in the case of lactoferrin than in the case of mesoglycan. Moreover, due to the presence of the second step, it is possible to obtain a slowdown of the release of significant importance, which is a crucial point for the targeted purpose. 

### 2.7. Cytotoxicity Test

**Figure 8:**
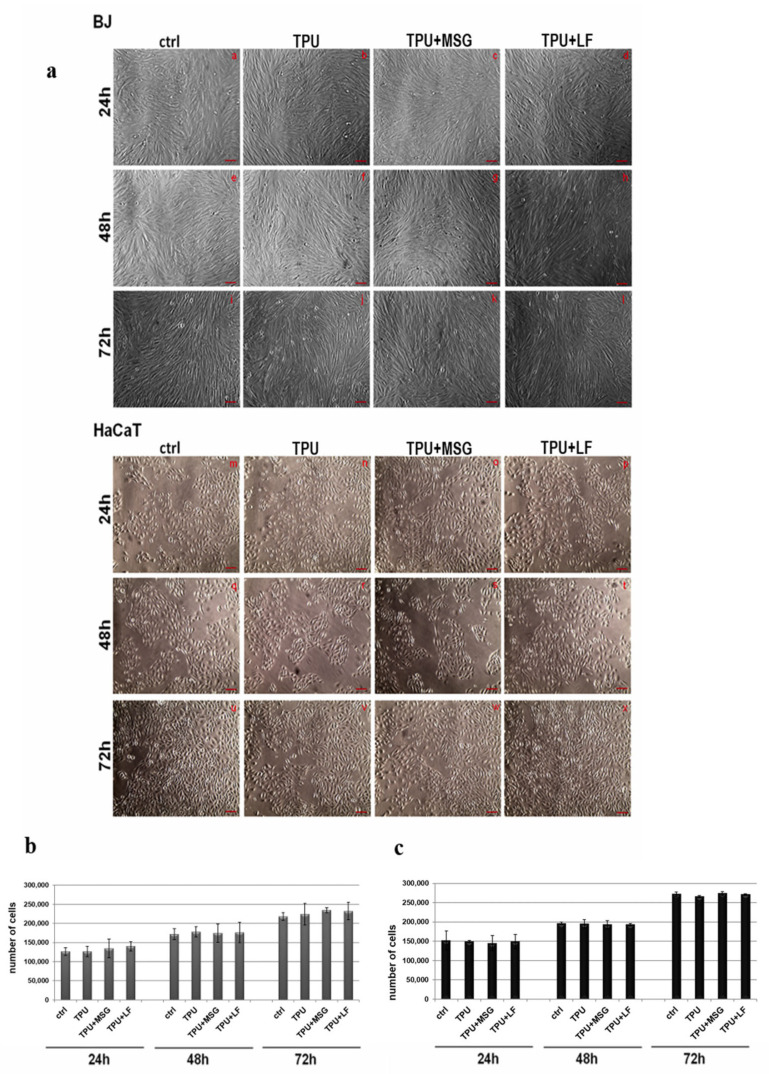
(**a**) representative images at 24, 48, and 72 h of BJ (panels a–d; e–h and i–l) and of HaCaT (panels m–p; q–t and u–x); scale bars equal to 100 μm. Hemocytometer cell counts of the same cells at 24, 48, and 72 h of contact with TPU, TPU-MSG, and TPU-LF in (**b**) fibroblasts and (**c**) keratinocyte growth medium. The data represent the mean of three experiments performed in triplicate with similar results.

All the polyurethane fibers appear compatible with cell viability. The in vitro compatibility of polyurethane fibers has been evaluated on HaCaT and BJ cell lines as human keratinocytes and fibroblasts, respectively. Figure 8a reports the representative images of cells in the presence of TPU, TPU-MSG, and TPU-LF at 24 h (panels a–d for BJ and m–p for HaCaT cells), at 48 h (panels e–h for BJ and q–t for HaCaT cells) and 72 h (panels i–l for BJ and u–x for HaCaT cells). No significant differences have been assessed when cells have been in contact with TPU, TPU-MSG, and TPU-LF compared to the non-treated experimental points. We further performed the hemocytometer count on both cell lines to confirm the absence of any cytotoxic effect. In the histograms in Figure 8b,c, the number of HaCaT and BJ cells, respectively, has been reported highlighting no notable differences in whether or not the cells were placed in the presence of the diverse kinds of TPU fibers.

Interestingly, the TPU fibers processed as described in this work appear compatible with cell viability. The cell lines analyzed here have been human keratinocytes and fibroblasts, representing the primary cell populations required in the skin wound healing process [37]. The in vitro preliminary tests we performed in this work first highlighted that the fibers do not induce any toxic action. Therefore, further studies are needed to prove the cell activation and the possible molecular events caused by mesoglycan and lactoferrin released from the PU fibers.

**Figure 9:**
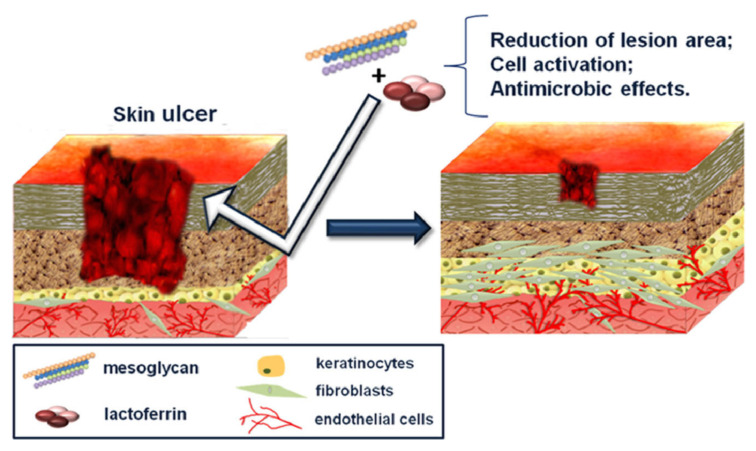
Schematic representation of the wound healing process using mesoglycan and lactoferrin. Reprinted with permission from [11]. Copyright © 2023 Elsevier.

However, it was previously demonstrated that MSG and LF favor the closure of skin wounds. The reduction of lesion area was evaluated by confocal analysis of mice biopsies underlying the correct tissue regeneration [11]. A schematic representation of the mechanism of wound healing is reported in Figure 9.

## 3. Materials and Methods

### 3.1. Materials

Thermoplastic polyurethane (TPU) was purchased from Lubrizol, OH, USA. N,N-Dimethylformamide (CAS: 68-12-2), and Tetrahydrofuran (CAS: 109-99-9) were purchased from Sigma Aldrich (St. Louis, MI, USA). Carbon dioxide (CO_2_, purity 99%) was supplied by Morlando Group S.R.L. (Sant’Antimo, Naples, Italy). Sodium salt mesoglycan (MSG) was purchased from LDO (Laboratori Derivati Organici spa, Vercelli, Italy); it consists of heparan sulfate (UFH-unfractionated heparin from 12 kDa up to 40 kDa; sulphurylation degree 2.6), heparin (40% low molecular weight in the range 6.5–10.5 kDa and 60% less than 12 kDa, sulphurylation degree 2.2–2.6) and dermatan sulfate, deriving from epimerization of glucuronic acid of chondroitin sulfate (molecular weight 18–30 kDa, sulphurylation degree 1.3) with a total sulphurylation degree equal to 9.1. Lactoferrin from human milk (LT) was provided by Sigma Aldrich (St. Louis, MI, USA). Sodium hydroxide (CAS: 1310-73-2), hydrochloric acid solution 37% v/v (CAS: 7647-01-0), urea (CAS: 57-13-6), and lactic acid (CAS: 50-21-5) were purchased from Sigma Aldrich (St. Louis, MI, USA). Sodium chloride (CAS: 7647-14-5) was purchased from Carlo Erba Reagents (Cornaredo, Italy).

### 3.2. Electrospinning

Polymeric solution for electrospinning was prepared as described hereafter: polyurethane (20% *w*/*w*) was dissolved in dimethylformamide/tetrahydrofuran solution (70/30 % *v*/*v*). The solution was stirred at 50 °C with a stirring rate of 300 rpm for 5 h. A 5 mL syringe pump was used in the electrospinning process. Optimized parameters are hereafter reported: voltage = 24 kV, flow rate = 1 mL/h, distance needle-collector = 23.5 cm, relative humidity = 35%, and temperature = 25 °C.

### 3.3. Supercritical Impregnation Apparatus

A homemade benchtop plant is employed to carry out the impregnation experiments. The experiments occur in a stainless-steel high-pressure vessel (NWA GmbH, Ahlen, Germany) with an internal volume of 100 mL, closed at the bottom and top with tight two-finger clamps. The CO_2_ is pumped to the vessel by a diaphragm piston pump (Milton Roy, mod. Milroyal B, Pont-Saint-Pierre, France) after cooling through a cooling bath. In order to guarantee the mixing inside the chamber, an impeller mounted on the top cap is used. The operating pressure is measured by a digital manometer (Parker, Minneapolis, MN, USA), and the temperature is determined by a K-type thermocouple with an accuracy of ±0.1 °C. To ensure the thermal control of the vessel, we used electrical controlled thin bands connected to a proportional–integral–derivative (PID) controller (Watlow, mod. 93, Toledo, OH, USA). A micrometric valve (Hoke, mod. 1315G4Y, Spartanburg, SC, USA) performs depressurization. A fixed quantity of active ingredients, MSG and LT, is loaded into a small container placed at the top and mounted axially on the impeller. The TPU film was set inside a paper filter on the bottom of the vessel. After closing the vessel using the finger-tightened clamps to reach the pressure and temperature chosen for the experiments, the CO_2_ was pumped, and the heating bands were activated. After this step, the CO_2_ delivery was stopped, the impeller was turned on, and the system was left in batch for the time chosen for the experiment. At the end of the time, a slow and controlled depressurization was carried out at a constant flow rate of 0.1 MPa/min. Finally, the impregnated sample was recovered and characterized once atmospheric pressure was reached. Each impregnation test was repeated three times; the difference between the tests was less than 5%, probably due to the possible difference during the depressurization phases, which allow the entrainment of the material through the filter paper and to the deposition of non-impregnated active ingredients on the film surface. 

### 3.4. Characterizations

A scanning electron microscope (SEM) coupled with an Energy Dispersive X-ray Spectroscopy (EDS) probe (Phenom ProX with EDS detector (Phenom-World BV, Eindhoven, The Netherlands)) was used for morphological characterization as well as analysis of elemental mapping. Before performing SEM analysis, the samples were coated with a thin gold film. Plot profiles were obtained from SEM images using the Plot Profile plug-in of Fiji software (version 2.9.0). The surface roughness parameters were evaluated through mathematical equations. Ra (arithmetic mean) and RMS (root mean square mean) were assessed using Equations (2) and (3):

(2) $$\mathrm{Ra}=\frac{1}{L}*\int_{0}^{L}Z\left(x\right)\mathrm{dx}$$

(3) $$\mathrm{RMS}={\left[\frac{1}{L}*\int_{0}^{L}Z{\left(x\right)}^{2}\mathrm{dx}\right]}^{0.5}$$

where Z(x) is the profile height function.

The liquid retention degree was evaluated by immersing a pre-weighed mass of mat in V = 25 mL in different solutions at room temperature (pure water, HCl solution (pH = 3), NaOH solution (pH = 13), and sweat simulant (pH = 5.5)). Sweat simulant was prepared according to EN 1811:2011; NaCl (10.8 g), lactic acid (1.2 g), and urea (1.3 g) in 1 L distillate water. The pH was adjusted to 5.5 by dropping the NaOH solution. After 24 h, the samples were re-weighed (M_eq_). The retention degree was evaluated according to Equation (4):

(4) $$Q\left(\%\right)=\frac{M_{0}-M_{\mathrm{eq}}}{M_{0}}*100$$

Contact angle (CA) measurements of specimens were analyzed by using a high-resolution camera. Droplets of liquid (100 μL) were dispensed on a 1 × 1 cm^2^ test sample. Five contact angle measurements were recorded. The contact angle was determined through the Drop Analysis plugin by using Image J software (version 1.53t). All results were reported as mean±standard deviation.

The amount of MSG and LT impregnated in the polymeric matrices was evaluated by UV–Vis spectrophotometry (model Cary 50, Varian, Palo Alto, CA, USA) at a wavelength of 206 nm and 250 nm, respectively. About 0.005 g of the film was put in 10 mL of PBS at 200 rpm and 37 °C. The MSG and LT loadings were measured by UV/vis analysis after 48 h of stirring, i.e., when the active principle was wholly released from the fibers. Using a calibration curve, the absorbance obtained was converted into concentration. The studies of release profiles were carried out at 37 °C using Franz’s cell, a static diffusion cell of 11 mL volume in glass, Type C (Hosmotic SRL, Vico Equense, Napoli, Italy) coupled to the UV/vis spectrophotometer used for the loadings. A PVDF membrane with a pore size of 0.45 μm was used to separate the donor chamber from the reception chamber. The receptor chamber was filled with PBS (pH 7.4) at 37 °C under constant stirring (500 rpm). Aliquots of 200 μL were withdrawn at fixed intervals and replaced with equal volumes of fresh PBS.

### 3.5. Cell Cultures

HaCaT cell line (Human immortalized keratinocytes) was purchased from CLS Cell Lines Service GmbH, and BJ cells (Human immortalized fibroblasts) were purchased from American Type Culture Collection (ATCC^®^ CRL2522™). Both cell lines were cultured, as reported by Belvedere et al. [8].

### 3.6. Hemocytometer Counting

The polyurethane fibers, containing or not containing mesoglycan or lactoferrin, were sterilized by UV irradiation by positioning them for 1 h under the light of a surface disinfection unit of the clean room. They were cut into pieces of approximately 0.8 cm^2^ and placed in the growth medium of the wells of a 12 multi-well plate in which 2 × 10^5^ fibroblasts/well and 1 × 10^5^ keratinocytes/well had been seeded the previous day. The bright field images were taken under an EVOS microscope (10× objective, Life Technologies Corporation, Waltham, MA, USA) after 24, 48, and 72 h. For further confirmation, at each experimental time, the cells were detached with trypsin; an hemocytometric count was performed by mixing equal volumes of 0.4% trypan blue (Sigma-Aldrich, St. Louis, MI, USA) stain with the cell suspension as reported in [6]. The Burker chamber with the trypan blue/cell mix was visualized under the optical microscope Axiovert 40 CFL (Carl Zeiss MicroImaging GmbH, Munich, Germany). To calculate the viable cells/mL, the average number of cells in one large square has been multiplied by the dilution factor (2) and then by 10^4^.

### 3.7. Statistical Analysis

Statistical analysis was carried out by performing a one-way ANOVA test. Tukey’s post hoc method was applied to analyze the significant differences between means (*p* < 0.05). Statistics software was used to analyze the experimental data. R^2^ coefficients were assessed to determine the goodness of the fitting processes.

## 4. Conclusions

Thermoplastic polyurethane polymeric fibers loaded with mesoglycan (MSG) and lactoferrin (LF) were fabricated by the electrospinning process and CO_2_-assisted impregnation under supercritical conditions to produce devices for wound healing treatment. The tests were conducted at a pressure of 150 bar and a temperature of 50 °C. The morphological analysis performed on the samples demonstrated that the chosen operating conditions do not modify the fibers, which, therefore, maintain the starting dimensions and orientation downstream of the loading of MSG and LF. The results obtained show that as the processing time increases, the quantity of mesoglycan and lactoferrin loaded increases; in particular, it emerged that after 48 h of the process, the amount of mesoglycan impregnated is 8% with respect to the total fed, the same percentage is obtained in 24 h in the case of lactoferrin. The advantages deriving from the impregnation of drugs on fibers are highlighted by the release tests conducted with the aid of the Franz cell and UV/vis spectrophotometry; in fact, in the case of drugs such as the graphs reach a plateau within a few hours, unlike the case in which they are loaded on the fibers thus favoring the achievement of a controlled and prolonged release over time. To use the devices produced, it is necessary to demonstrate their biocompatibility; for this reason, cytotoxic tests have been conducted to evaluate the effect of the TPU, TPU+MSG, and TPU+LF systems on fibroblasts and keratinocytes, cells responsible for the generation of granulation tissue in the wound healing and essential for re-epithelialization. No toxic events emerge from the tests, making them promising for the application of interest, which can be tissue engineering, wound healing, and drug delivery.

## Figures and Tables

**Table 1:** Kinetic constants obtained from Ho’s model.

Sample	K_1_ (g/g^×^h)	K_2_ (g/g^×^h)
MSG pure	4.46	-
LF pure	0.77	-
MSG-TPU	1.89	0.01
LF-TPU	4.93	0.32

## Data Availability

Not applicable.
